# Miscellanies

**Published:** 1836-04-01

**Authors:** 


### MISCELLANIES.

### Cyclopædia of Anatomy, Part V.

### Anatomical Plates of Mr. Quain.

The press of matter in the present number prevents our fully noticing these as well as some other works, for which this must be the apology. In our next number we shall bring up our arrears, and adopt a plan which will enable us to give a more full account of *all* new works, than has hitherto been possible.

We may observe, in passing, that both the works, whose titles are before us, have high claims on the attention of students. They should both be in the possession of all who hope to prosecute their anatomical studies with advantage and success.

---

### Reclamation.

*To the Editors of the Medico-Chirurgical Review.*

Gentlemen,—In the course of the Review, given in your last number, of my work on "Spinal Irritation," I observe that you put the following query:—"Has Dr. Marshall never seen reason to suspect in these cases"—(viz. those simulating diseased heart)—"that the symptoms arose from *Masturbation?*" To this I can decidedly reply in the negative. I never had, in any of my *published cases,* the shadow of reason to suspect the presence of such a vice. A vice which, when it has gone to so great a degree as to produce symptoms, such as those detailed in my cases, I have almost uniformly found accompanied by a decay of the mental as well as the physical powers. Moreover, I regret to say, that, in all my experience of this loathsome habit, I have very rarely succeeded, *even under the strictest surveillance,* to effect a cure; but, on the contrary, have found the miserable victims dwindle into a state of fatuity, hopeless mania, or a premature death.

In case the third, there was not the slightest ground for suspicion, that such a habit existed. In case fourth, I may say the same. The subject of this, subsequently became, for six months, an inmate in my own family. A more estimable and strictly moral youth I have rarely known, and from childhood he had been distinguished for the same unblemished regularity of conduct and feeling. Permit me to add, that the subjects of both these cases are now *remarkably well grown and robust young men.*

I observe in your remarks on the cases of simulated phthisis, that you are inclined to consider case the FIRST, as one of "Hooping Cough." By the testimony of her then medical attendant, the patient had had well marked hooping cough at four years of age. Since I received your Review, I have applied to her surviving relatives, and they *decidedly* corroborate this statement. I may here remark, that I have noted this hacking convulsive cough as a frequent symptom in spinal irritation. I observe that Dr. Griffin makes the same remark.

I hope, Gentlemen, that neither you nor the public will suppose that I make this reply in a contentious or querulous spirit. Nothing can be further from my feelings. With the most profound indifference to "ephemeral opinions," I hold such gentleman-like and generous criticism as that which you have bestowed upon my unpretending work, to be the very life and health of our science, and our professional literature.

I am, Gentlemen,
With sincere respect, yours, &c.

JOHN MARSHALL.

*Manchester, Feb.* 12*th,* 1836.

---

DISEASE OF HEART, LIVER, &c. By JAMES JOHNSON, M.D.

Single cases are not often useful, unless they bear upon some obscure or disputed point in pathology, diagnosis, prognosis, or therapeutics. The following case is not without interest in some of these respects.

It is now about three years since a young gentleman, a patient of Mr. Chinnock's, came under the observation of the narrator. Mr. Barnes, then aged about fourteen years, was born in Bencoolen, and came to this country when about six or seven years of age. He was, for some years, at an academy near London, and appeared to enjoy good health, being active and cheerful. He returned from school to the house of a most indulgent and friendly protector at Kensington, some months before Dr. Johnson saw him, and was under the care of Mr. Chinnock, who treated him for affection of the heart and disorder of the digestive organs.

On examination, at the time mentioned above, it was ascertained by auscultation, that hypertrophy of the heart, to a considerable extent, had taken place, and that a dropsical tendency was commencing. Even then (and the phenomena became more intense afterwards)—even then, the action and sounds of the heart were frightful. The *bruit de scie* and the *bruit de soufflet* were often loud and distinct; but, in general, there was a tumultuous noise and action, resembling the churning of milk. The heart beat over a large space, and there was great epigastric pulsation. The carotids pulsated largely, and emitted a loud bellows-sound. He could only lie with his head well raised, and his breathing was easily rendered difficult by any exertion. The urine was scanty, and the secretions depraved. Dropsical symptoms now became prominent, and diuretics, with mercury, sometimes reduced the swellings of the lower extremities—sometimes only kept them at bay—but at length failed, and effusion was but too evident, both in the abdomen and chest. The liver, too, was found to be enlarged.

No medical man, of experience, could look at this poor youth, with organic disease of the heart and liver —with legs ready to burst—abdomen fluctuating, and with one side of chest at least (the right) containing water—with the kidneys refusing to act—the intestinal evacuations highly disordered and the patient himself scarcely able to breathe in the semi-erect posture—no man could contemplate this picture, and venture to give the slightest hope of recovery to the friends—or even that life could be protracted for some weeks.

As the whole class of diuretics had been tried, one after another, without making any impression on the urinary organs, that channel of evacuation was given up, and elaterium, with calomel, was exhibited. The effect was surprizing—and was often afterwards witnessed. It soon cleared out the whole of the water from the cavities and cellular membrane—and enabled the kidneys to act by means of the mildest diuretics. Again and again the symptoms returned; and as often were they removed by the same means. The disease of the heart remained, of course, in statu quo.

After one of the clearings out by the elaterium and mercury, Nature appeared to make a vigorous effort against fearful odds—the dropsical effusions did not return—the appetite became good—the urine natural—and the secretions healthy. He now shot up in growth so fast, that every month he appeared to be an inch or two taller. He even assumed a healthy aspect of countenance—and could walk some miles without inconvenience. All this time, the heart presented the formidable or rather frightful phenomena described at the beginning of the case.

He determined on a profession, and embraced that of medicine. He was articled to Mr. Chinnock, his own medical attendant, who now became his master. This was on the 31st March, 1835. The improvement in his general health was for the first four months most remarkable, he took fish alternate days only; he religiously avoided animal food, and lived principally on vegetables and farinaceous food. He grew very considerably during this period, and a corresponding development was observable, the mind was remarkably active. He entered into the mysteries of the profession with much enthusiasm, the physical signs of disease still continuing, although not so strongly marked.

In the month of October of last year, the inconvenience of the heart began again to tell, and he was finally obliged to leave his kind and indulgent preceptor, and take refuge, once more, under the roof of Mrs. Hurley—a lady who, in the absence of father and mother, had proved herself to be more than both to this amiable youth—nursing him day and night, till the last day of his existence. The dropsical swellings returned again—the breathing became distressing—and all remedies failed. The legs were punctured by Mr. H. J. Johnson—and, for one or two nights, he slept comfortably. The dyspnœa again became pressing, and again the legs were punctured; but with little relief. He expired, after great sufferings, on the 13th December.

The body was examined on the day after decease, by Mr. Henry James Johnson, in the presence of Mr. Clifton, Mr. Chinnock, and Dr. Johnson. The appearances were noted by Mr. Johnson, as follows:—

### Post-Mortem Appearances.

The lower extremities were œdematous to a considerable degree.

Thorax. In the *right* pleural cavity there was a large quantity of serous effusion of a dark colour, without albuminous concretions, or any decided traces of recent inflammation. The lung was in some degree compressed by the effusion, but exhibited no material alteration.

The costal and pulmonary pleuræ on the *left* side were closely united by almost universal adhesions, the cellular character of which shewed that they had existed for some time. The lung exhibited nothing remarkable.

The cavity of the pericardium was obliterated by the general and intimate adhesion of the two layers of that membrane. The adhesions were mostly cellular and extremely close.

The heart itself was enormously enlarged, and, as usually happens, rounded in its form. The cavities on the *right* side were dilated, with but little hypertrophy of their parietes. The *left auricle* was in a similar condition. The *left ventricle* was both dilated and hypertrophied, the hypertrophy being principally marked in the thickness of the ventricular septum, and the magnitude of the columnæ carneæ.

The valves, and the great vessels were unaffected with any appreciable alteration.

*Abdomen.* There was a moderate quantity of dark serum in this cavity.

The liver was very large, but apparently not altered in its structure. The spleen was rather large also. The kidneys were in a natural state, as were the remaining contents of the abdomen.

Upon minute inquiries, it could not be discovered that this young gentleman had ever suffered from acute rheumatism, or had ever presented any symptoms of pericarditis. Yet the pericardium was universally adherent to the heart by ancient connexions. The same was found to be the case with Mr. M'Kerrell, the unfortunate gentleman who poisoned himself by prussic acid in Regent-street. He never had had rheumatism, or any symptoms indicative of pericarditis.

In the case of Master Barnes, might not the progressive enlargement of the heart itself, cause, by stretching, friction, and distention, this same adhesion? The not very unfrequent occurrence of pericardial adhesion, when not suspected, suggests the propriety of leeching and counter-irritation in hypertrophy of the heart, where no inflammatory symptoms are present.

Even the temporary recovery from such an alarming condition as this young gentleman was in, may teach us to be guarded in our prognosis, where there is youth on the side of the patient.

The strikingly beneficial effects of the elaterium and mercury, are worthy of commemoration; for at all times, it is desirable to relieve the urgent symptoms; and it is of great consequence to prolong life for a certain period, when we have no hope of curing the disease.

There is a lady now residing in Howland-street, aged 73, or perhaps more. She has had immense hypertrophy of the heart, with valvular disease, for many years. Two years ago, when the reporter first saw her, she was dropsical in chest, abdomen, and extremities. She has been repeatedly cleared of the effusions by elaterium and mercury, when all the more efficient diuretics had failed. The disease of the heart remains, of course; but its effects, by which effects life is generally terminated, and always rendered miserable, are removed, from time to time, and the old lady gets so well as to indulge freely in the pleasures of the table, and even to mix in society. Then she abandons physic, and returns to Bromley, her usual residence. The dropsical effusions recur, and she comes to town, wondering that the doctor can only make a temporary, and not a permanent cure of her complaint! The wonder, on the part of the doctor, is, that even a temporary truce, at her time of life, and under existing circumstances, can be obtained, with such a formidable enemy!

In this case, as in the case of Master Barnes, the removal of the dropsical effusions by elaterium and mercury, invariably restore, for a time, the functions of the kidneys.

N. B. She was visited by me this week, and is so very well that she intends to return to the country forthwith. The irregularity of action in the heart is less than it has been for years—her breathing is free—and all dropsical symptoms have disappeared. She takes an eighth of a grain of elaterium, with two grains of calomel, twice a week. This medicine produces three or four copious watery stools, and keeps her nearly free from complaint.

J. J.

---

### MEDICAL POLITICS.

We do not often take up the passing events of the day, because they are much better adapted to the weekly journals than to a trimestral publication. Where the subjects, however, are of great and permanent importance, we have not hesitated, as our readers know, to express our sentiments, and that with equal freedom and independence.

Three minor subjects have, for some time past, excited a good deal of attention in the medical world—viz: Pauper Practice—Coroners' Inquests—and Life-Insurances. We shall venture a few, and but a few remarks on each of these topics.

### I. Pauper Practice.

The profession has lately been rather scandalized by the exposures that have been made of its poverty. The pauper population is being farmed by the paupers of the profession. The *latter* sympathizing with the distresses of the *former*, and being, moreover, imbued strongly with philanthropy and political *economy*, have generously undertaken to attend parish paupers at a rate so low, that the annual sum for medicine and attendance, would not pay for one half of the snuff or tobacco used by the said paupers! When the parish, or rather the union of parishes, is put up — not to the highest, but to the lowest bidder, there is seldom or never a lack of contracting parties. The lowest is consequently taken by the Guardians or the Poor-law Commissioners, and then there are numerous remonstrances made through the medium of the daily and weekly press, against the hard-heartedness of the Commissioners and the meanspiritedness of the successful candidates.

After paying some attention to this subject, we have come to the conclusion that the charges against both the above parties are very much exaggerated — and, moreover, that they are not always made with perfect freedom from motives of self-interest that will not bear a very rigid investigation. In respect to the Commissioners and Guardians, we really do not know in what other way they are to proceed, than by *public contract* — unless it is by the old way of *private jobbing*. If, indeed, they gave the contract to *any person* who offered the lowest tender, without official proof of qualification, then we would hold up our hands and voices against such procedure. But if, on the other hand, they admit tenders from none who are not legally qualified to practise by the established institutions of the country, we cannot conscientiously blame them for accepting the lowest scale of remuneration.

It will be said—it has often been said, that this system will induce many young gentlemen, fresh from the schools, to accept the low reward, in order, first to get experience, and ultimately an introduction to private practice in the unions. Granted. Is this not the case with nine-tenths of those who give their *entirely gratuitous advice and attendance* to the poor in our innumerable Dispensaries, Hospitals, and Eleemosynary Institutions?—For what do they offer *their* services—even without the 2s. 6d. or 3s. 6d. per head per annum? First, for experience—secondly, for introduction or publicity — and thirdly and principally for MONEY—the necessary consequence of the first two! We put it to the heads and the hearts of every one of our readers, whether or not this statement be correct. Not a single individual, we venture to say, can deny its truth. Then what becomes of the cry against those young men, who having expended large sums on their education, but being without capital to purchase practices or partnerships, accept of three or four shillings per head per annum, for attending the sick poor of a union — not with any prospect of *immediate* profit from the contract, but with the hope of an introduction—experience—and ultimately a settlement in the said union.

This introduction of a stranger into a parish or union of parishes is, we acknowledge, very unpleasant, and even detrimental to the resident practitioners. But how is it to be avoided? We have repeatedly adverted to the overcrowded ranks of the profession — and we ask those who advocate an increase of facilities in the education of medical practitioners, what is to become of the NEOPHYTES, if they are to be browbeaten and reviled for undertaking the drudgery of practice at a rate of compensation, which will scarcely supply them with bread and cheese, during the first years of their labours? These medical GUARDIANS of the profession are like so many FRANKENSTEINS, who first create beings, and then cast them on the world, not only without provision, but with persecution and punishment!!

A plausible objection, indeed, is made to these parochial NEOPHYTES, that they are without *experience*, and consequently that they must gather it among the poor. This is the case, God knows, with more than the parochial NEOPHYTES!! Experience must be acquired somewhere—and if a young man's chief hopes rest on the success of his practice among the humbler classes of society, we do not see any valid objection to this mode of debut, *after a proper education.* But here we would ask a somewhat searching question. If there be a lack of *experience* on the part of the young practitioner, fresh from the schools and hospitals, is there not a lack of *time*, on the part of the old practitioners, who, while they have charge of parishes, have also the charge of a large circle of private patients? What is the consequence? Why that many of the paupers must often be attended by pupils, who have little or no medical education at all!

Considering the extent of the medical curricula at present insisted on, we are very far from stigmatizing the *inexperience* of those who have complied with the prescribed regulations, and undergone the proper examinations. We very much doubt whether the pauper—perhaps even the opulent patient, would not be as safe in the hands of a modern ELEVE, who had just left the wards of an hospital, and the dirty work of dissection, as in the hands of an old parish doctor, of the "*Ancien Regime.*"

We have adverted to the disinterestedness of these complaints against the "UNIONS of PARISHES," and the arbitrary divisions made by the Commissioners. Let us take a passage from one of the loudest of the complainants, Mr. Edward Manby, of East Rudham, Norfolk.

"Thus, if parishes A. B. or C. lie in a division marked by the Board as a Western division, and the person who, for many years has been the attendant on such parishes, happens to reside on the Eastern side of the line, those parishes are unceremoniously assigned to another practitioner, who happens to contract for the Western division, *thus depriving the original attendant not only of his parish attendance, but necessarily of a large portion of his casual private practice, owing to his not visiting such parishes so frequently as when in attendance on the poor.*"*

Here, then, we have the real state of the case—the real objects and ends of pauper attendance, let out inadvertently! Private and *emolumentary* practice is, in all cases the final object of *gratuitous* or parochial attendance! This is the fact, divested of all special pleading. We would, therefore, advise all our provincial brethren to be cautious in their appeals on these *tender* subjects, convinced, as we are, that they will not further their interests by such procedure, as we shall presently shew.

It is impossible, according to our view of the question, to condemn the contract for a *low rate* of attendance on the poor, without condemning also the *gratuitous* attendance on the same classes, in large towns, by the medical officers of our hospitals, infirmaries, and other charitable institutions. This cheap and gratuitous devotion of time and talent, hurts, no doubt, the private practitioner. But the state of society requires — and, we believe, the ultimate good of the community is promoted by the system in question. These, our opinions, are unbiassed and independent. We have nothing to do with pauper practice or parochial guardians or Poor-law Commissioners, and therefore we can *reason* on the subject rather than declaim.

We think there can be but one opinion on this principle, *that the highest qualification and the lowest expense,* should guide the choice of the medical attendant on the poor. The scale of remuneration is the lowest that can well be, and yet the provincial practitioners complain that numbers of medical men — qualified by law, are ready to close with contracts, by which their own private practice is injured in consequence of the introduction of strangers into their neighbourhood. Well, what do they pray for? An augmentation of the medical remunera-

* Lancet, 30th Jan. 1836, p. 711.

tion! What will—what *must* be the inevitable consequence of such an augmentation? Why, that the Commissioners and Guardians would be besieged by candidates for employment. Aye—and by men of first-rate education, equal, or indeed superior to the education of the physician and pure surgeon of the present day! But, if the very highest grade of education were objected to, and *experience* required, would that be wanting among the candidates? Not at all. There are more army and navy medical officers, now on half-pay, than would supply all the unions in England!

If, therefore, the higher rate of emolument be conceded, by petition to the Legislature, the question will then be—not who is the lowest bidder, but who is the best qualified? And do the provincial surgeons imagine that any one of *them* will afterwards be appointed by the Commissioners and Guardians? If they do, they calculate without their host! The said Commissioners and Guardians, being first irritated against the local practitioners, and then supplied with ample lists of able and well-educated candidates, will *naturally*—perhaps, we ought to say, *properly* appoint those, who are unencumbered with private practice, to attendance on the poor.*

Thus, then, the provincial practitioners are steeping a rod in pickle to whip their own backs. By any petition to Parliament for increased remuneration, they will induce hundreds and thousands of a redundant medical population to rush through the portals of preferment, in order to catch at the prize, small as it may be! And thus, into every union in the kingdom, will be introduced a stranger, either of excellent modern education (a young man), or an experienced practitioner, from the long lists of half-pay medical officers, who will unquestionably, in a very few years, cause the provincial surgeons to sup sorrow for their announced appeal to the Legislature!

But, it may be asked, would not an augmented scale of remuneration, and the appointment of highly-educated or long-experienced men, for the specific purpose of parochial practice, be beneficial to the pauper population itself? We answer, that we believe it would. And if the provincial surgeons have no other object in view than to ameliorate the condition of the poor, *by deteriorating the condition of themselves*, why then we admire a liberality and disinterestedness which we so rarely meet, and advise them to proceed!

We have thrown out these observations from an impartial consideration of the question. We leave them for reflection to those whom they most concern. We declare, once more, that we have no self-interest in the matter, and that we are anxious only to direct the judgment of our brethren into a proper channel.

### II. Coroner's Inquests.

It is not our intention to discuss the propriety or impropriety of medical coronership. We believe that a medical man, if he studied *law* as well as physic, would make a better coroner than the mere law coroner, as at present constituted. But if the coroner were a surgeon, a medical man must, after all, be called in to give evidence on oath to the jury—for surely no one will contend that the coroner himself should proceed to open bodies or analyze the contents of stomachs, and then return to the court and decide the points by his own *ipse dixit*. No. There must be *another* medical man, and the jury are bound to go by *his* evidence, rather than by the opinion of the coroner. We see this every day. In the case of the unfortunate Mr.

* Be it remembered that, by the present plan, the lowest bidder (provided he is legally qualified) can claim the contract, and the Commissioners have no choice. But, if the scale of remuneration be fixed, the choice of candidates inevitably falls to the commissioners or guardians, and the establish ed practitioners of the union will have no power to prevent the introduction of a stranger, by accepting the contract themselves!

Mc Kerrell, who committed suicide in Regent-street, Mr. Gell, the Coroner, charged the jury, not in direct terms, but by indirect insinuations, to bring in a verdict of FELO-DE-SE, against the evidence of Dr. Johnson, who swore that he believed the deceased laboured under temporary insanity. Fifteen of the jury gave their verdict according to the evidence of Dr. J. and five, according to the leaning of the coroner.

Since, then, there must be a medical *witness* in most inquests, would it not be better that a medical man, well qualified in all medico-legal inquiries, should be appointed by the Crown, to superintend the examination of all bodies, and assist the coroner with his advice and testimony on all occasions? This appears to us to be the wisest plan, and most accordant with the spirit of law and justice.

This, however, is not the question. We believe a petition to Parliament is in the course of signature, praying for compensation to medical men, who are called upon to give evidence in Coroners' Courts, for the loss of their time. We have taken a rather unpopular view of the *pauper question*, but here we fully agree with the petitioners in the propriety of their application to Parliament—though we doubt much whether the "collective wisdom" will deign to listen to the just prayers of the petitioners. Members will be so immersed in fierce political contests, that useful bills will, in many cases, be cushioned. Still the attempt ought to be made.

Medical men, however, have considerable power in their own hands, on this point. A physician or surgeon, is bound like any other member of the community, to give evidence of such matters as come under his cognizance—and that without any recompence for loss of time. This is a duty which we all owe to law, justice, and the good of society. A surgeon is called in to a man who is shot in a duel, and dies of the wound. He is bound to give his testimony, without remuneration, like any other witness. He is paid, or supposed to be paid for his professional attendance on the patient, and for the judicial attendance in court, we do not believe he can claim any remuneration. But if the coroner desires him to proceed and examine the body, POST MORTEM, the case is entirely altered, and the surgeon may refuse to do so, without the slightest risk of penalty. In the *first* instance he is a witness of what he has seen, and he is bound, like any other witness, to give his evidence for nothing. But in the *second* instance, he is *employed* by the Coroner to do work—to investigate a case—to furnish the Court with the result of that work, and he is just as much entitled to remuneration as the Coroner himself, or his clerk who takes down the depositions. This important distinction ought to be carefully made in any petition to Parliament, otherwise the prayer will be damaged—and the defect detected by some of the legal members of the senate. The faculty should claim no compensation for parole or circumstantial evidence respecting any thing which fell under their observation casually, and *anterior* to the inquest. But they may fairly claim remuneration for every thing done by the request of the Coroner and the Court. We are quite sure that the Coroner cannot insist on any work or attendance of this kind—and therefore the medical man may refuse to do it, unless he is compensated for his time and trouble.*

### III. Insurance Offices.

We observe that several members of the profession are turning their thoughts towards remuneration for answering the queries of LIFE-ASSURANCE COMPANIES. Perhaps this subject is not well considered—and we are confident that it has not been clearly expounded. A man thinks it desirable to insure his life for the benefit of his family. To effect this, he is bound to produce credible evidence, first of his moral character and habits—and, secondly, of his state of health. For the first species of evidence he refers to a friend—say

* Since the above was written, Mr. Wakley's bill seems likely to be successful.—Ed.

his solicitor: for the second, to his usual medical attendant. It is the general custom for the applicant to wait upon these two referees, or to write to them, soliciting the favour of their answering the queries of the insurance office. Now here the favour is clearly conferred on the patient and client—not on the office. And if the doctor thinks himself entitled to a guinea, surely the solicitor or friend may claim a similar remuneration. No one of our readers suffers more loss of time—and that when it can ill be spared—than we do in this way; but we can see no remedy or recompence—save the sense of obligation which may remain in the patient's own breast.

The insurance office cannot be expected to pay for documents which the proposer himself must produce as the basis of the transaction. The office has a physician and surgeon of its own to pay, who examine the individuals at the office, if in town—and the Company have to pay a guinea on every policy in the country, to their own medical agent. It cannot be supposed that they will agree to pay additionally for the evidence which the proposer himself is bound to furnish.

But there is a fatal objection to the proposal, which has not presented itself to the minds of those who advocate the payment of a guinea to the medical referee—it opens a door to fraud. There are frauds enough practised on insurance companies, without opening out an additional channel. There are unprincipled men in all professions—in physic, law, and perhaps even in divinity. Two people—say an attorney and a doctor—combine to tax the various insurance offices for their own benefit. A., the attorney, proposes a real, or perhaps an imaginary life, for assurance at the Rock or the Crown. He refers to Mr. B., the medical attendant of the proposer. Mr. B. states *in confidence* to the board, that the life to be insured is not a good one, as the patient had burst a bloodvessel some time previously, and is disposed to consumption. The fee is, of course, cheerfully paid, and the Company congratulate themselves on having escaped a bad insurance, through the *candour and honesty* of the medical referee. Inviolable secrecy is preserved by the office. Such a scheme might be carried on for years, with the various insurance offices in London, and that with very little chance of detection!

When we first heard of the question, the possibility of such a contingency as the foregoing occurred to us. And soon afterwards, when we heard the matter discussed at an insurance office, we were a little surprised to hear one of the ablest ACTUARIES in Europe declare his conviction, that such swindling transactions would be the inevitable result of a preliminary fee to a referee.

Before we quit the subject, we have a little bit of advice to give to our brethren, founded on no very limited range of observation, and knowledge of human nature and the world as it is. The medical profession has always been allowed, even by its enemies, to be an extremely *liberal* one, especially as respects pecuniary compensation. It is universally admitted, that the medical man is the only professional man who cheerfully dedicates his time and talents, *gratuitously*, to the relief of the poor—and that he is ever ready to succour distress, even among the *apparently affluent*, when he finds them labouring under the complicated miseries of sickness and pecuniary difficulties. This favourable impression on the public mind should be fostered rather than dissipated. There are many prejudices *against* the profession; but the foregoing one, in its favour, redeems many a fault, real or imaginary! We regret to see a disposition springing up, to place the medical man on a PAR with the LAWYER and PARSON in respect to *legal* emoluments. Such a system will, most assuredly, deteriorate the medical character in public estimation. There is no rule but that of conscience and humanity to guide the medical practitioner. The patient is at the mercy of the physician or surgeon. He may kill or cure him! This, at least, is the general opinion. Nothing can be more conducive to the reputation of the faculty, or the prosperity of the individual member, than a conviction, on the part of the community, that the safety of the patient is paramount to all other considerations—particularly of a pecuniary nature—in the mind of the practitioner.

The trifling advantages that may be gained by *legal exactions*, will never compensate for the loss that will be occasioned by popular prejudice. Many and many a time have we been actually and unequivocally cheated out of our fees and compensations; but we never dreamt of going to law on these occasions. There is a maxim, "that he who once *injures* you will afterwards hate you." This, we believe, does not hold good in respect to *cheating*. We have often traced some of our most valuable connexions to the recommendations of those who acted unjustly towards us themselves!

There are some, no doubt, who have amassed large fortunes in practice, and who have had the character of being mercenary jews throughout life. They are dangerous examples to follow. Perhaps they would have done still better, had they imitated the liberality of a Cline, a Baillie, a Babington, or a Cooper. These men, we believe, never hunted after fees, or squeezed every guinea that they possibly could out of the pockets of their patients. As honesty is the best policy in the common concerns of life, so liberality is profitable, *cæteris paribus*, in professional avocations. So at least, we have found it.

---

### Metropolitan University—Medical Reform.

It is not very long since we were subjected to unmeasured ridicule, for suggesting the idea of one governing Body, (by whatever designation characterized,) empowered to regulate the curricula of medical education—examine the candidates—and confer diplomas.* The plan then projected does not appear so unfeasible now, as it was two years ago. Something of the kind seems to be in contemplation by Government, while, strange to say, those contemporaries who ridiculed us, at that time, now claim the merit of having always advocated the measure in question! Yes. There are conformers in medical, as well as in general politics—men who anathematise any measure that is unlikely to take place—especially if it militates against their interests or inclinations; but, who veer round with the tide of circumstances, and boldly proclaim themselves the advocates of that which they formerly denounced!

Our bitterest adversaries have never insinuated that we had any personal motives, either of ambition or lucre, in the course which we have pursued in medical politics. To us it is perfectly immaterial, whether Whig or Tory—aristocratic or popular principles, gain the ascendant. Our interests are totally distinct from, and independent of, medical politics. This is now so well known, that we defy the breath of censure to impugn the statement. But we claim the liberty of advocating those principles which we sincerely deem to be just—and the more so, as the advocacy cannot be deemed mercenary.

Knowledge, as far as medicine in concerned, is of two kinds—distinct in their nature, and still more so, as regards the mode of their acquirement. The one is *general*, or literary and scientific—the other is *professional*. The first is preliminary, and includes languages, mathematics, and the elements of useful knowledge—the other is medical, which includes, of course, the whole range of professional studies—anatomy, pharmacy, therapeutics—clinics—and the various other branches of the healing art. The preliminary education may be acquired any where—in the back attic of a private house, as well as in the "academic bowers and learned halls" of a university. Hundreds of individuals have become profound linguists and mathematicians, without any tuition at all—merely by their own application to books. The professional education, on the contrary, can *only* be learnt (in a private manner at least) from those who

---

* Medico-Chirurgical Review, April, 1834.

have previously studied it—and *only* in certain places, where the means of teaching it are amply collected, and arranged.

The *preliminary* branch is distinguished from the *professional,* by another characteristic feature. It can be tested accurately by a public or private examination. The other cannot be so tested, as will be presently proved.

Now, if these propositions be founded in truth, and they will hardly be disputed, it follows, according to reason, justice, and sound policy, that the *source,* or the *means* of acquiring the preliminary education, especially of medical men, should form no part of inquiry—but only the *amount* of that knowledge, seeing that it can be acquired anywhere, and that its amount can be ascertained with the greatest precision, by the test of examination. Would it not—will it not be a sad blot on the liberality and intelligence of the nineteenth century, if it be determined that Greek, Latin, and mathematics, if learnt at Harrow, Westminster, or any other seminary of education, shall not be considered as good—*if of the same amount*—as that which is acquired in the neighbourhood of Somerset Place, or St. Pancras? Will not such a distinction be a plain indication that the legislator aims not so much at the actual possession of the *preliminary* education, as at the power of conferring a monopoly on particular schools? The inference is as clear as the sun at noon-day?

A rumour (we hope unfounded) prevails, that such a distinction will be made —and consequently that a monopoly will thus be secured to the present LONDON UNIVERSITY and KING'S COLLEGE!* If such an invidious distinction—if such an odious mark of favouritism should be established by LAW (for JUSTICE is out of the question) under the auspices of a Government distinguished from all preceding governments, for enlightened views and liberal sentiments, it will be an anomaly that can only be accounted for by want of reflection on the subject, or by treacherous and designing councillors! Such a procedure will be reprobated by ninety-nine of every hundred medical men in the United Kingdom—a class of His Majesty's subjects which is neither destitute of intelligence nor influence—even in political matters. The physician and surgeon have access to all ranks and parties—and that, at times, when reason is listened to more calmly than at most others—on the bed of sickness. Such a partial, unjustifiable, and invidious piece of legislation, as the above, would rankle in the breast of every liberal member of the profession, and would soon be manifested in the senate.

Even if it were desirable that the two colleges in question were recognized as the only seminaries qualified to turn out candidates for the Bachelorship of Arts, still it would be a great injury to the schools of medicine in the metropolis to render such a degree imperative on the medical student—and for this obvious reason, that the two colleges are *also* medical schools, and would thus have an immense weight of favour thrown into their scale. The only way to avoid this glaring injustice would be to insist on a good classical and mathematical education on the part of the medical aspirant, leaving him the option of acquiring

---

* What liberal mind can fail to grieve at seeing the dying embers of bigotry blown upon by certain portions of society—as at Oxford and King's College! Will it be believed that the sapient big-wigs of the *latter* establishment have objected to Professor Ritchie, Dr. Webster, and Mr. Kiernan, becoming candidates for medical and philosophical professorships—on account of their RELIGIOUS CREEDS!! An infidel or an Atheist, who would subscribe to the infernal code of his Satanic Majesty just as freely as to the 39 Articles, would be received as a candidate, at King's College, without the slightest objection. But a conscientious believer in Christianity is pushed from the arena—because his creed differs, on some mysterious and incomprehensible points, from the orthodox code!! Shame on those who vainly attempt to roll back the tide of religious liberty from the nineteenth to the ninth century!

that education where he pleases. Even on this liberal basis, there will still be a considerable advantage on the side of the two colleges, on account of their being general as well as medical schools.

The PROFESSIONAL EDUCATION stands on quite a different footing. It cannot be properly attained through the medium of a private tutor, or by any, even the most extraordinary application to books. It can only be acquired in the lecture-room—the dissecting-room—and the wards of a public hospital. It cannot be *completely tested* by any examination before a Board of Examiners. IT REQUIRES ADDITIONAL CHECKS and AUXILIARIES. From no limited observation and experience, we are convinced that the channels of PROFESSIONAL KNOWLEDGE should be clearly and distinctly marked out by the Legislature—and that authentic and honest proofs should be adduced by the candidate for medical diplomas, that the said professional information did flow through these RECOGNIZED CHANNELS. Here we make our stand. Here we join issue with our ultra-liberal contemporaries.

The only CONDITION on which a medical school should be *recognized*, appears to be this—ITS CAPABILITY OF AFFORDING ADEQUATE MEDICAL INSTRUCTION, as determined by a liberal and honourable Board. We have heard it urged, indeed, by some of our contemporaries, and by men whom we greatly esteem, that the source and means of PROFESSIONAL acquirements, should not be inquired into, and that the only test should be the EXAMINATION. These men, we firmly believe, are deceived—or have not a sufficient knowledge of the subject. We would ask these men how a student can be *properly* tested by an examination in anatomy, chemistry, surgery—and more especially in CLINICAL knowledge by such examination? It is utterly impossible! We grant, indeed, that *examinations* might be made much more efficient and searching than they now are; but still they would be grossly imperfect, without the aid of other checks, which will be presently pointed out. A student may, by dint of hard study, and the aid of a GRINDER, be capable of describing the origin and insertion of every muscle—the ramification of every artery—and the distribution of every nerve—and yet, if taken into a dissecting-room, he might be scarcely capable of distinguishing the sciatic nerve from the sartorius muscle! He may be able, from books and grinding, to lay down the diagnosis, prognosis, and treatment of every disease in Good's Nosology, and yet, if taken into the wards of an hospital, he might not be capable of distinguishing small-pox from measles. This is no caricature. The Editor of this Journal knew an instance of a young gentleman, some 37 years ago, who, without ever having attended a single lecture—without ever having had a dissecting-knife in his hand, passed an examination in LINCOLN'S-INN-FIELDS, with such eclat, that he was called back into the Board-room, and publicly complimented on his great proficiency in anatomy and surgery! This was at a time when testimonials of attendance on lectures were not demanded for "surgeon's mates," as they were then called, in the Navy. True, this was a remarkable, but by no means a *singular* case.*

We are well aware, at the same time, that the "CERTIFICATE SYSTEM," as it is called, is a very fallacious one—and that dunces, drones, and idlers are every day *certified* as industrious, and well-qualified pupils. This is the *abuse*, and not the *use* of the system. It may be made a very efficient system, by proper regulations. But an abolition of this same "certificate system," bad as it is, would convert every Grinder's garret into a school of medicine, and a memory crammed with technical terms would too often be substituted for sound, but laborious knowledge.

---

* For the credit of this gentleman, he quickly left the service—took eagerly to his studies—and afterwards distinguished himself as a surgeon in this country.

The primary and most important tests must ever be in the lecture-room, the dissecting-room, and the wards of an hospital. And the instructors in these places can be very effectually prompted to the task of regularly examining their pupils at stated periods,—simply by non-recognition of the certificates from those who refuse to comply with this regulation. The tutors of King's College, and of many other seminaries of instruction, examine their pupils at stated epochs, and record their progress, and regularity of attendance. Why should not medical tutors, who are far better paid, adopt the same course? It must come to this at last. These minor public examinations would have another good effect. It would train the pupils for a still more important and public examination at the close of their studies.

In respect to the RECOGNITION of medical schools, much need not be said—because much has been said to no good purpose. If a proper system of examinations be established, the minor and inadequate schools will soon be extinguished by a natural law. They will not sink by *gravity;* but they will float on the surface, like bubbles, and soon burst, by the breath of public opinion.

Now let us, for a moment, discuss the subject of PUBLIC EXAMINATIONS. We have conversed with many of the most distinguished physicians and surgeons, on this point, and the all but universal answer has been—"Oh! the nerves of *English* pupils would never stand the brunt of a PUBLIC EXAMINATION." The blood has boiled, with indignation, in our veins, at hearing this declaration;—and we have, more than once, retorted, in sharp language, that *English* nerves, and English moral and physical courage, are, at least *equal* to the same nerves and courage in other countries, where these examinations are public! We have heard this enunciation from the mouths of surgeons who have fearlessly stood to their duty under showers of balls from Napoleon's victorious troops among the mountains of Spain,—and on the plains of Waterloo! No. The British candidate will not shrink from a PUBLIC EXAMINATION; but the superannuated EXAMINER will endeavour to throw the cloak of cowardice over the pupil whom HE is afraid to examine in public! This is the plain *English* of the matter. Where do we find, in this world, people more tender of the reputation of their neighbours, than of their own? No where? In a PUBLIC examination, the fairness or unfairness of questions is submitted to the general sense of the profession; and if the examiner comes to the arena, CRAMMED with minute and *specific* puzzlers, which he has previously drawn from *books,* he will be scouted, as he well knows, by any candid listener. And here we would ask why the examination of medical students should be conducted on principles differing from those which are adopted at our most celebrated universities?—Why should not a series of written questions be submitted to the candidate to fill up, as he best can, in a room where he shall have no access to books or men? It could not be said, in this case, that the EXAMINEE would be terrified by a public examination. This would, most undoubtedly be the fairest and the surest way of guaging the amount of a candidate's knowledge—and it would be attended with this advantage, that no cavil or quibble, could be afterwards made use of, by the rejected candidate or the examiner. There the questions and answers stand on record, and, if demanded, they can be published in an authentic form. It is a hypocritical mockery of justice to tell a rejected pupil that a *viva voce* examination will be published,—if he particularly desire it. By whom is it published? By the examiner himself, who would thus have the power of shaping and modifying the questions and answers according to his memory, or rather his inclinations! There can be little doubt that in such a procedure, the examiner wold evince a wonderfully rententive memory of all the *fair* questions which he put to the Neophyte, and of the *faulty* answers that were returned;—perhaps, too, his recollection would prove sadly treacherous in respect to the reverse of the picture! Why should not all this be obviated by the LITERA SCRIPTA—the written questions and answers? We can imagine no valid reason against this plan, especially as it is sanctioned by experience in both Oxford and Cambridge.* Nay, we would go farther. We would insist on medical teachers producing, with their certificates, a *tentamen* of this kind, on a smaller scale, executed in the schools of medicine.

And here we would demur, most conscientiously, to the validity of those objections which are strenuously urged against the admission of hospital physicians and surgeons, as well as lecturers, into the composition of the examining body. Such an exclusion would be highly detrimental to the interests of science. The men thus proscribed are those who are best qualified to test the candidates in the minute elementary knowledge which alone such candidates can be expected to possess. The very circumstance of being engaged, for years, in extensive private practice, would incapacitate the most talented private practitioner for the task of examining minutely into the acquirements of the candidates in anatomy, chemistry, botany and other branches of elementary knowledge. If the examinations were conducted in public, or in the written documentary manner which we have described, no unfair play, personal jealousy, or partiality could possibly influence the acceptance or rejection of the candidate. But while we would not exclude these public functionaries, we would include a fair proportion of private practitioners to be present at the decision, and join in the examination, if they pleased. It would, indeed, be monstrous to exclude a man from *testing* elementary knowledge, *because* he is in the daily habit of *teaching* it in all its minutiæ!

Many conjectures are afloat, and many schemes have been broached, as to the organization of the projected Metropolitan University. We have a right to offer our plan, as well as our neighbours. It is true we have not much voice in the state—but we have lived long enough to be able to form some idea of the wants as well as the wishes of the profession.—Were we called upon for an opinion—of which there is extremely little probability—we would thus sketch the outline of the projected establishment.

We would suggest that TEN of the most distinguished men in each of three present departments of the profession should be selected by the Crown—the THIRTY to form the "SENATUS MEDICUS ACADEMICUS." Six of these—two physicians, two surgeons, and two apothecaries—to form a sitting committee, for organizing the establishment, drawing up rules and regulations, &c. Half of the SENATUS to go out every three years, their places to be filled up by the Crown—or by election. The SENATUS ACADEMICUS to hold a Court of Examination every three months—viz: once a week, for three weeks, at the beginning of each quarter, in order to effect three examinations of each candidate.

The above, we think, might form the basis, or skeleton of the organization, leaving all details to the "collective wisdom" of the SENATUS. We conceive that a triennial elevation of fifteen members of the medical profession to the highest honours of medical science, would be a greater stimulus to laudable ambition than any which now exists.†

* While writing these lines, we were informed by one of the most influential members of the Council of the College of Surgeons, that the Court of Examiners of that Body, is meditating on the plan in question—the examination by written questions and answers. We need hardly say that we were not a little gratified by this intelligence.

† As the honour would be great, the emolument should be very moderate. It might be advantageously regulated by the principles adopted at Public Boards, such as those of Insurance Companies—"namely, no work, no pay." Those only should receive any compensation for their time and labour who dedicated these to the actual working of the machinery.

But it may be asked, if such a scheme were to be put in execution, what is to become of the three existing corporate bodies in this metropolis? If filthy lucre be their claim to life—buy them up, or pay them off. Let every existing corporator have, during his life, an income out of the new revenues derived from examinations, equal to their average income from the present system. Let the Fellows of the College of Physicians be paid a sum equivalent to their present profits—we imagine that such a sum will not impoverish the state!—The same with the other bodies. They all say that their duties are heavy labours and responsibilities, for which they have no adequate remuneration.

If these Bodies continue to execute their present functions, then we say that the erection of a METROPOLITAN UNIVERSITY, will be a work of supererogation, and a needless expense to the state as well as a burthen on the profession. If an examination by the SENATUS ACADEMICUS does not entitle a man to practise his profession in this country—if he must afterwards go to the College of Physicians, and the Apothecaries' Company, for another examination and a License —then we say that such a MONSTROUS ANOMALY never was erected in any age or in any country!!

It is to be recollected, however, that there is now no real *compulsion* to pass the ordeal of the College of Physicians and the College of Surgeons. The Charter of the first is obsolete by time—and that of the other has no other power than the power of OPINION. The College of Surgeons is the only *surgical* tribunal—and its testimony is the only one which a medical practitioner can adduce, as a passport to the higher grades of surgical practice. The student consequently resorts to it. But if a HIGHER tribunal be erected, who, we ask, will consult the *inferior?* The Society of Apothecaries, then, is the only *legal* stumbling-block to the new SENATUS. But we have seen that Chartered Corporations have been swept away, not by tens or twenties, but by hundreds. And we see no reason why one single, self-elected, pharmaceutical corporation which, by accident, has usurped the highest legal control over the profession, should be suffered to resist the current of improvement and reform.

We have always admitted that the Apothecaries' Company has done more good, and has advanced the qualifications of the general practitioner higher than either or both of the other two Corporations—by gradually extending the period and amount of medical studies. For this they deserve the highest praise. But we contend that the power of doing this good has been vested in the wrong place. It ought to be entrusted to one supreme and paramount tribunal—not to one which superintends a single department of the profession. We claim an equality—perhaps a superiority over other countries in intelligence—arts—and science. And yet we are infinitely inferior to them in the organization of those institutions which direct this intelligence—as far, at least, as medical science is concerned!

We think, indeed, that it is high *time* that the multitudinous, discordant, and unequal medical institutions of this great country, should be incorporated into one uniform, efficient, and liberal SENATE, empowered to legislate for the United Kingdom—Scotland and Ireland, having, of course, their separate senates, but only as branches of the parent institution, and acting in strict conformity with it.

What modifications of the present order of things in the profession may result from the New University, and Mr. Warburton's Bill, we have no means of judging. The following extract from a paper published just two years ago, contains suggestions which subsequent reflection has not induced us to alter.

"*First,* we would suggest that no one should enter on the career of his medical studies before the age of eighteen, when, if designed for general practice, he should undergo an examination in classical and general knowledge prior to his apprenticeship. This apprenticeship we would limit to two years—especially in the country, where lectures could not be attended. At the age of twenty, his general medical studies should commence, and be pursued through three academic years in any recognized school within the limits of Great Britain, allowing one or two years of that time, if desired, to be spent in any proper foreign school. At the age of 23, a public examination in medicine, surgery, midwifery, pharmacy, &c. should be passed before a faculty or board, composed of physicians and surgeons. This rigid tentamen over, he should have a diploma or, degree conferred on him, with the designation of 'ORDINARY PHYSICIAN,' entitling him to practise any or all branches of the profession *ubique terrarum*, or at least throughout the British dominions.

*Secondly*, those who aimed at a higher grade, and who wished to earn it by a more extended and expensive education, general and medical, might be indulged—and possibly without disadvantage. Such aspirant should not be allowed to enter on the professional curriculum till the age of 20, at which period he should undergo a strict examination in Greek, Latin, mathematics, and general knowledge. His medical curriculum should embrace six, or at least five, years of clinical and preceptorial studies, two of which might be allowed to foreign schools. His examination should be the strictest of all, and in public, after which he might go out either in physic or surgery, as he pleased—and have a degree, conferring on him the title of 'GRADUATE PHYSICIAN' in the one case, or 'DOCTOR in SURGERY' in the other—or, if both, 'DOCTOR in MEDICINE and SURGERY.'

Thus there would still be two, if not three designations; but the education of all would experience a very considerable approach to amalgamation. The 'ORDINARY PHYSICIAN' should always have the option of passing into the class of PHYSICIAN EXTRAORDINARY, or GRADUATE, by addition of study, or rather by undergoing the examination imposed on the latter originally.

As men may work with their own tools, so the ordinary physician might be permitted to furnish his own medicines to his own patients, but not to sell them, and not to charge for them as a whole, but as a part of the remuneration for skill and attendance. This will be the most difficult point to settle, whether by the Legislature or the individual practitioner. We are as firmly convinced as ever, that the general practitioner, or, as we propose to designate him in a new order of things, the PHYSICIAN in ORDINARY, will never attain the height of respectability and of satisfaction in his avocations, till he can charge *entirely* for his skill, without having any thing to do with the actual dispensing of medicine. But, as opinion runs contrary to this, especially in the country, let opinion have its way. A day will come, we apprehend, when the practice of physic and surgery will be entirely independent of the practice of pharmacy. Till then, some improvement may, perhaps, be made on the present plan. Suppose the legislature were to enable the general practitioner to charge from 3*s*. 6*d*. to 5*s*. for his daily attendance, or, where daily attendance is not necessary, for his separate visits—with a margin of 1*s*. 6*d*. to 2*s*. 6*d*. for medicines furnished, according to the nature of the case;—we think that such a scale of remuneration would be infinitely preferable to the present one, where the whole expense of a sickness comes in at the end of the year, in the form of a bill of parcels, throwing the medical skill and attendance entirely out of sight, and merging all in the gross, and often nauseous, *materiel* which the patient has swallowed during his confinement!!"*

We would still adhere to the above suggestions, and we submit them respectfully, but confidently, to the Government, to Parliament, and to the forthcoming SENATUS MEDICUS LONDINENSIS.

---

* Med. Chir. Review, April, 1834.

